# A EUFOREA comment on a lost comorbidity of asthma

**DOI:** 10.1186/s13223-023-00816-0

**Published:** 2023-06-30

**Authors:** Diego M. Conti, Peter W. Hellings, Zuzana Diamant, Leif Bjermer, Milos Jesenak, Vibeke Backer, Wytske Fokkens, Susanne Lau, Elizabeth Van Staeyen, Glenis K. Scadding

**Affiliations:** 1 The European Forum for Research and Education in Allergy and Airway Diseases Scientific Expert Team Members, Brussels, Belgium; 2 KU Leuven Department of Microbiology and Immunology, Allergy and Clinical Immunology Research Unit, Leuven, Belgium; 3 Clinical Department of Otorhinolaryngology, Head and Neck Surgery, University Hospitals Leuven, Leuven, Belgium; 4 Department of Otorhinolaryngology, Laboratory of Upper Airways Research, University of Ghent, Ghent, Belgium; 5 Department of Otorhinolaryngology, Academic Medical Center, University of Amsterdam, Amsterdam, The Netherlands; 6 Department of Respiratory Medicine & Allergology, Institute for Clinical Science, Skane University Hospital, Lund University, Lund, Sweden; 7 Department of Respiratory Medicine, First Faculty of Medicine, Charles University and Thomayer Hospital, Prague, Czech Republic; 8 Department Clinical Pharmacy and Pharmacology, University of Groningen, University Medical Center Groningen, Groningen, Netherlands; 9 Department of Pulmonology and Phthisiology, Department of Pediatrics, Department of Clinical Immunology and Allergology, Jessenius Faculty of Medicine in Martin, Comenius University in Bratislava, University Hospital in Martin, Martin, Slovakia; 10 Department of Otorhinolaryngology, Head & Neck surgery, and Audiology. Rigshospitalet, Copenhagen University, Copenhagen, Denmark; 11 Department of Otorhinolaryngology, Amsterdam University Medical Centres, Amsterdam, The Netherlands; 12 Department of Pediatric Respiratory Medicine, Immunology and Critical Care Medicine, Charité Universitaetsmedizin Berlin, Berlin, Germany; 13 Department of Allergy & Rhinology, Royal National ENT Hospital, London, UK; 14 Division of Immunity and Infection, University College, London, UK

**Keywords:** Asthma, Comorbidities, Allergic rhinitis, Chronic rhinosinusitis with nasal polyps, Chronic rhinosinusitis without nasal polyps

## Abstract

“Epidemiology of comorbidities and their association with asthma control” (Tomisa, G., Horváth, A., Sánta, B. et al. Epidemiology of comorbidities and their association with asthma control. *Allergy Asthma Clin Immunol* 17, 95 (2021). 10.1186/s13223-021-00598-3) is an interesting paper reflecting data collection from more than 12,000 asthmatic patients in Hungary regarding their condition and associated comorbidities. We found it valuable that the paper provides an overview of asthma comorbidities not usually considered in similar reports. Nevertheless, we believe that chronic rhinosinusitis (CRS) with or without nasal polyps (CRSwNP or CRSsNP) should have been listed due to its high incidence and prevalence, its association with asthma which is also endorsed in both GINA and EPOS, as well as in several peer-reviewed scientific papers, and to reflect the role of this comorbidity in poor control and a most severe presentation of asthma for the patient. Consequently, several targeted therapies (especially monoclonal antibodies) used for several years in severe forms of asthma are now indicated also for the effective treatment of nasal polyps.

## Main text

The paper by Tomisa et al. [1] describes the prevalence of several common comorbidities of asthma in relation to patient characteristics (age, gender and body mass index [BMI]) and their association with asthma control in a large, specialist-managed patient population.

Although just cryptically mentioned in this paper and presumably hidden under the name of seasonal rhinitis/perennial rhinitis (according to ARIA intermittent or persistent), CRS is a common and prevalent disease imposing a significant health problem that is known to affect up to 10.9% of the global population, while severe CRSwNP is present in approximately 1–2% of the European population [2]. The prevalence of asthma in CRSsNP patients is 21.2%, and 44.9% for CRSwNP patients [3]. Both lead to a significant burden on society in terms of healthcare consumption and productivity loss [4–6].

CRSwNP is an invalidating disease characterized by type 2 inflammation with a major impact on quality of life of those severely affected and often associated with comorbidities such as asthma, allergies, sleep-disordered breathing and non-steroidal anti-inflammatory drugs exacerbated respiratory disease (N-ERD) [7, 8].

Despite a substantial burden and affect of quality of life in individuals (extensively confirmed with validated questionnaires including the general health EQ-5D [9, 10] and SF36 [11, 12]), their relatives, as well as society and health economics, CRS often remains un-diagnosed, and thus under-estimated, and consequently under- or mistreated [13].

The work by Tomisa et al. does not give a prominent role to rhinitis or CRS to influence the control or severity of asthma. However, given the anatomic, epidemiologic and pathophysiologic connections between the upper and lower airways [14–16], the concept of united airway disease (one airway – one disease, as exposed in EPOS and GINA) has gained more interest in the past years, leading to better diagnosis and therapeutic approaches in patients with such problems [17–19]. Lower airway inflammation often co-exists in CRS, with up to two-thirds of those patients affected by comorbid asthma, COPD or bronchiectasis [20–23].

Several immune mechanisms have been reported to be involved in the naso-bronchial interaction in patients with global airway disease. Type 2 inflammation, present in the majority of adult asthma patients, has also been demonstrated in their sinonasal secretions [24, 25]. In addition, challenging either compartment of the airways has been shown to induce inflammation in the counterpart [26].

More recently, targeted treatment options with biologics provided further insight into the underlying mechanisms and relationship between upper and lower airways in patients with asthma and CRSwNP. Therefore, it is not surprising that biological treatments targeting inflammatory pathways and molecules such as IL-4, IL-13, IL-5 and IgE in both upper and lower airway T2-high inflammation are effective in both asthma and CRSwNP [27–31].

Further evidence for associations between upper and lower airway compartments has shown that co-existent CRS increases the patient´s risk of exacerbations even if they have few asthma symptoms, and it is associated with a more severe asthma course, especially in patients with nasal polyps [32, 33].

Like other comorbidities such as obesity, sleep apnoea, and anxiety, the presence of AR or CRS are among the criteria for difficult-to-treat asthma [34].

In fact, the HELIUS study showed CRS to be associated with adult-onset asthma [35] and the Asthma Clinical Research Network demonstrated that CRS is associated with increased risk of asthma exacerbations [36].

Chen et al. [37] identified patients newly diagnosed with asthma in Taiwan and analyzed the incidence of CRS in that population. After adjustment for gender, age and medical comorbidities, they showed that asthma is an independent predictor of CRS, with or without nasal polyps (OR: 2.58 for CRSsNP).

In addition, endoscopic sinus surgery in asthma has been reported to improve multiple clinical asthma parameters [38]. The frequency of severe asthma exacerbations decreased in 84.8% (95% CI, 76.6–93.0%) of patients and the number of hospitalizations decreased in 64.4% (95% CI, 53.3–75.6%). Decreased use of oral corticosteroids was seen in 72.8% (95% CI, 67.5–78.1%) of patients; inhaled corticosteroid use decreased in 28.5% (95% CI, 22.6–34.5%) and bronchodilator use decreased in 36.3% (95% CI, 28.9–43.7%) of patients [39].

Unable to access the questionnaire applied, we can only speculate why CRS is not explicitly mentioned in this paper. Possibly, the sample consisted of patients with allergic asthma, which is associated with AR more than CRS and not with late-onset non-allergic asthma.

However, we found it very interesting that this work is based on a previous one (Tomisa G et al. Prevalence and impact of risk factors for poor asthma outcomes in a large, specialist-managed patient cohort: a real-life study. *J Asthma Allergy*. 2019; 12:297–307. 10.2147/JAA.S211246), in which the same sample was used, and AR and CRS are named as the first comorbidity found and also remarked as one of the main risk factors for uncontrolled disease.

## Data Availability

Not applicable.

## Abbreviations

AR: Allergic rhinitis
CRS: Chronic Rhinosinusitis
CRSsNP: Chronic Rhinosinusitis without Nasal Polyps
CRSwNP: Chronic Rhinosinusitis with Nasal Polyps
N-ERD: NSAIDs Exacerbated Respiratory Disease
NSAID: Non-steroidal anti-inflammatory drug
COPD: Chronic obstructive pulmonary disease
EPOS: European Position Paper on Rhinosinusitis and Nasal Polyps
GINA: Global Initiative for Asthma
